# Microglial activation and blood–brain barrier leakage: chicken and egg?

**DOI:** 10.1093/brain/awab149

**Published:** 2021-05-24

**Authors:** Paul Edison

**Affiliations:** Department of Brain Sciences, Faculty of Medicine, Imperial College London, London, UK

## Abstract

This scientific commentary refers to ‘Microglial activation and blood–brain barrier permeability in cerebral small vessel disease’ by Walsh *et al.* (doi:10.1093/brain/awab003).

**This scientific commentary refers to ‘Microglial activation and blood–brain barrier permeability in cerebral small vessel disease’ by Walsh *et al.* (doi:10.1093/brain/awab003).**

Cerebral small vessel disease (SVD) is a significant contributor to cognitive dysfunction and disability in older people. However, there is no specific treatment available to prevent the progressive deterioration of cognitive function in patients with cerebrovascular disease and cognitive impairment. Neuroinflammation has been implicated in the pathogenesis of sporadic SVD. It has also been suggested that altered innate immunity is related to SVD progression.^1^^,^^2^

Cardiovascular disease, such as hypertension and diabetes, represents a major risk factor for the pathological processes associated with SVD, which include increased blood–brain barrier permeability and neuroinflammation. Studies have shown increased blood–brain barrier leakage in patients with stroke, white matter hyperintensity and vascular cognitive impairment.^3–5^ It has been suggested that circulating biomarkers of both inflammation and endothelial activation are increased in SVD, and may predict the progression of white matter hyperintensities.^6^^,^^7^ Chronic inflammation can lead to white matter hypoperfusion and hypoxia, which can trigger the release of metalloproteins that disrupt the extracellular matrix of the vascular endothelium, leading to opening of the blood–brain barrier. This implies that blood–brain barrier permeability and microglial activation may be related.^8–10^

In this issue of *Brain*, Jessica Walsh, Hugh Markus and colleagues^11^ evaluate whether blood–brain barrier permeability is increased—and how this may relate to white matter hyperintensities—in patients with sporadic SVD or with the monogenic form of cerebral SVD, ‘CADASIL’ (cerebral autosomal dominant arteriopathy with subcortical infarcts and leukoencephalopathy).

The study included 20 CADASIL patients, 20 sporadic SVD patients and 20 control subjects. All individuals underwent PET and MRI in a single session using a PET magnetic resonance scanner. The translocator protein PET radioligand ^11^C-PK11195 was used to measure microglial activation, while dynamic contrast-enhanced MRI (DCE-MRI) was used to assess blood–brain barrier permeability. In addition, white matter hyperintensities—considered a marker of SVD—were quantified on FLAIR images by a single trained rater using the semi-automatic contouring technique. Lacunes were defined as CSF-filled cavities at least 3 mm in diameter, and permeability maps for DCE-MRI were created using Patlak graphical analysis with sagittal sinus input. ^11^C-PK11195 binding was analysed using a simplified reference tissue model incorporating a correction for vascular binding. Walsh *et al*.^11^ were able to include 17 control subjects, 16 sporadic SVD patients and 14 CADASIL patients in the final analysis.

The results showed that blood–brain barrier permeability was increased in normal-appearing white matter in the sporadic SVD group compared to the controls, whereas there was no statistically significant difference in the CADASIL group. In addition, the volume of ^11^C-PK11195 binding was significantly higher in sporadic SVD compared to controls in both seemingly normal white matter and white matter hyperintensities. In the CADASIL group, ^11^C-PK11195 binding did not differ significantly versus controls, whereas white matter hyperintensity was increased. Walsh *et al*.^11^ further applied principal component analysis for 93 blood markers relating to cardiovascular disease inflammation and endothelial activation for each participant in the sporadic SVD group. Both mean and hotspot volumes of blood–brain barrier permeability were associated with white matter hyperintensity and seemingly normal white matter, whereas there was no association with ^11^C-PK11195 binding. In the CADASIL group there were no such associations.

The authors use the term ‘hotspots’ to delineate volumes of increased microglial activation and blood–brain barrier leakage. White matter hyperintensity was measured using a semi-automatic quantification manually drawn by a single trained rater, but inter-rater reliability was high (0.98–0.99). Lacunes were also defined manually. To create the tissue maps, Walsh *et al*.^11^ used 3 mm erosion from both sides (from CSF and also from grey matter). While this takes into account the CSF signals and spillage of signals to the other tissue, it may have some impact on the volume of white matter hyperintensity, especially in the periventricular area. However, as the authors used the same maps to evaluate blood–brain barrier leakage and microglial activation in the same regions, this should not greatly influence the results. The authors used the sagittal sinus instead of the arterial input, which may have some effect on the absolute quantification. However, as the authors point out, this is a well accepted method.

In their analysis, the authors divided voxels into four different groups: those with both microglial activation and increased blood–brain barrier leakage relative to controls, those with microglial activation but not increased blood–brain barrier leakage, those with increased blood–brain barrier leakage but not microglial activation, and those with neither. They conclude that there is increased blood–brain barrier permeability and increased microglial activation within white matter in sporadic SVD and that regions of increased blood–brain barrier permeability do not overlap with regions of increased microglial activation, implying that these are spatially distinct processes. In CADASIL, by contrast, they show increased microglial activation, but no increase in blood–brain barrier leakage. They therefore conclude that both blood–brain barrier leakage and microglial activation play a role in SVD, but that blood–brain barrier leakage is less important in CADASIL. This is an important observation, but it must be considered in the context of the small number of subjects in the study.

The findings also imply that blood–brain barrier leakage and microglial activation can independently influence the pathogenesis of sporadic SVD. Consistent with this, in sporadic SVD, the endothelial activation inflammation markers played a significant role in the increased white matter permeability and volume of the permeability hotspot. Additionally, one could argue that different individuals may have different susceptibility to inflammatory cytokines and other insults in different parts of the brain.^12^

Important issues that remain to be addressed are (i) how to predict susceptibility to persistent blood–brain barrier leakage; (ii) whether a subacute insult can predispose to blood–brain barrier leakage; and (iii) whether there is any spontaneous repair of the blood–brain barrier after a period of time. These issues could not be explored in the current cross-sectional study. Microglial activation may be very dynamic and, while there are regions of microglial activation without white matter hyperintensity, it is difficult to establish whether microglial activation has ‘burnt-out’ in those regions. It would be interesting to evaluate whether microglial activation appears first and can lead to white matter hyperintensity. Walsh *et al*.^11^ have already demonstrated that there is increased microglial activation in seemingly normal white matter, and it would be worthwhile examining whether these regions go on to develop white matter hyperintensity.

Walsh *et al*.^11^ found microglial activation and blood–brain barrier leakage to be spatially distinct in their cross-sectional study. However, previous preclinical work has found neuroinflammation and blood–brain barrier leakage to be spatially related.^8^ It is possible that these two processes could occur independently of each other. Equally, it is possible that microglial activation may be followed by white matter hyperintensity, or vice versa, and that this could even be related to the type of insult or the time at which the insult occurs. To test this possibility, a longitudinal study with multiple time points would ideally be required. However, conducting a multiple time point study in a short span of time, particularly with PET, would be challenging.

While CADASIL has a distinct pathological process and molecular mechanism compared to sporadic SVD, it is entirely possible that blood–brain barrier leakage may not be the primary event in CADASIL. There was no association between blood markers of cardiovascular dysfunction and blood–brain barrier permeability within the CADASIL group, again suggesting a distinct molecular mechanism, although the numbers were small. Walsh *et al*.^11^ also found evidence of increased microglial activation in CADASIL, which is consistent with other neurodegenerative and neuroinflammatory processes and implies that blood–brain barrier leakage is not necessary to initiate microglial activation.

Walsh *et al*.^11^ recruited patients with genetic and sporadic forms of SVD, which are both well characterized disorders. However, longitudinal data would be valuable in informing us about the primary aetiology of the disease process and could help reveal whether the processes of microglial activation and blood–brain barrier leakage occur independently of each other, or whether microglial activation precedes or follows white matter hyperintensity in SVD. The observation of microglial activation in normal-appearing white matter suggests that microglial activation may very well precede white matter hyperintensity, which in turn implies that agents targeting inflammation and systemic cytokines could have a therapeutic effect.
